# Contribution of cell wall peroxidase‐ and NADPH oxidase‐derived reactive oxygen species to *Alternaria brassicicola*‐induced oxidative burst in *Arabidopsis*

**DOI:** 10.1111/mpp.12769

**Published:** 2019-02-08

**Authors:** Evelin Kámán‐Tóth, Tamás Dankó, Gábor Gullner, Zoltán Bozsó, László Palkovics, Miklós Pogány

**Affiliations:** ^1^ Plant Protection Institute, Centre for Agricultural Research Hungarian Academy of Sciences H‐1022 Budapest, Herman Ottó út 15, Hungary; ^2^ Szent István University Faculty of Horticultural Science H‐1118 Budapest, Villányi út 29‐43, Hungary

**Keywords:** *Alternaria*, *Arabidopsis*, cell wall peroxidase, ERECTA, JAR1, NHO1, VIGS

## Abstract

Cell wall peroxidases and plasma membrane‐localized NADPH oxidases are considered to be the main sources of the apoplastic oxidative burst in plants attacked by microbial pathogens. In spite of this established doctrine, approaches attempting a comparative, side‐by‐side analysis of the functions of extracellular reactive oxygen species (ROS) generated by the two enzymatic sources are scarce. Previously, we have reported the role of *Arabidopsis* NADPH oxidase RBOHD (respiratory burst oxidase homologue D) in plants challenged with the necrotrophic fungus *Alternaria brassicicola*. Here, we present results on the activity of apoplastic class III peroxidases PRX33 (*At3g49110*) and PRX34 (*At3g49120*) investigated in the same *Arabidopsis*–*Alternaria* pathosystem. ROS generated by *Arabidopsis* peroxidases PRX33 and PRX34 increase the necrotic symptoms and colonization success of *A. brassicicola*. In addition, the knockdown of *PRX33* and *PRX34* transcript levels leads to a reduced number of host cells showing an extracellular burst of ROS after inoculation with *A. brassicicola*. Our results also reveal an age‐dependent transcript distribution of ROS‐producing peroxidase and NADPH oxidase enzymes, and some potential new components of the RBOHD, PRX33 and PRX34 signalling networks.

## Introduction

Plant cells respond to invading microbial pathogens with a series of changes in the activity of their various metabolic pathways. One of the most apparent cellular reactions on pathogen attack is a timely, coordinated accumulation of reactive oxygen species (ROS) in the apoplast and in some cellular compartments, which is often referred to as the ‘oxidative burst’. ROS that are released during the course of the oxidative burst include the free radicals superoxide (O_2_^•^¯), hydroxyl radicals (^•^OH) and nitric oxide (^•^NO), with unpaired valence electrons, as well as the non‐radicals hydrogen peroxide (H_2_O_2_) and singlet oxygen (^1^O_2_) (Groß *et al*., 2013; Mignolet‐Spruyt *et al.*, 2016; Mittler, 2017). Several enzymatic systems have been recognized as cellular sources of the oxidative burst in the apoplast of plant cells. These include NADPH oxidases, cell wall peroxidases (class III secretory plant peroxidases), amine and polyamine oxidases (AOs and PAOs), oxalate oxidases and quinone reductases (Kärkönen and Kuchitsu 2015). Endeavours to decipher the roles of NADPH oxidases and cell wall peroxidases in plant diseases and immunity have led to significant discoveries, but the functions of AOs, oxalate oxidases and quinone reductases are still largely elusive.

The most extensively studied sources of extracellular ROS in plants challenged by pathogenic microbes are respiratory burst oxidase homologue (RBOH) NADPH oxidases localized in the cellular plasma membrane (Frederickson Matika and Loake, 2013; Liu and He, 2016). The genome of *Arabidopsis thaliana* (hereafter referred to as *Arabidopsis*) encodes 10 RBOH proteins. Of these 10 isoforms, *RbohD* and *RbohF* are transcribed at the highest levels in root and leaf tissues (Torres *et al.*, 1998). The contribution of *Arabidopsis* NADPH oxidases RBOHD and RBOHF to the apoplastic oxidative burst, host immunity and/or cell death regulation has been reported in a wide range of *Arabidopsis*–pathogen interactions. These interactions include virulent or avirulent bacterial, oomycete or necrotrophic and biotrophic fungal pathogens of *Arabidopsis* (Marino *et al.*, 2012). Concerning the significance of apoplastic *Arabidopsis* class III peroxidases in plant–pathogen interactions, PRX33 and PRX34 (encoded by loci *At3g49110* and *At3g49120*, respectively) have been described as sources of an extracellular oxidative burst when plants/cell suspensions are challenged by avirulent strains of *Pseudomonas syringae* (Bindschedler *et al.*, 2006) or treated with microbe‐associated molecular pattern (MAMP) elicitors (Daudi *et al.*, 2012; O’Brien *et al.*, 2012). The proportions of ROS released by NADPH oxidases and peroxidases were evaluated by a pharmacological approach. In *Arabidopsis* cell suspensions treated with various MAMP elicitors, at least 50% of the H_2_O_2_ produced could be credited to peroxidases, predominantly to PRX33 and PRX34, and the remaining 50% or less was attributed to NADPH oxidases and intracellular sources (O’Brien *et al.*, 2012). Peroxidases PRX33 and PRX34 were found to contribute to resistance against virulent and avirulent strains of *P. syringae*, as well as against infections caused by *Botrytis cinerea* and powdery mildew fungi (Bindschedler *et al.*, 2006). Interestingly, an oxidative burst triggered by the treatment of *Arabidopsis* plants with a cell wall elicitor prepared from the fungus *Fusarium oxysporum* could be abolished by knocking down the transcript levels of *PRX33* and *PRX34* through the transgenic expression of a French bean class III peroxidase cDNA (*FBP1*) in an antisense orientation (Daudi *et al.*, 2012). This genotype has been referred to as *asFBP1.1*. The depletion of the *PRX33* mRNA level in an *Arabidopsis* T‐DNA insertion line, however, resulted in reduced susceptibility to *F. oxysporum*, indicating that PRX33 promotes susceptibility to this particular fungus (Lyons *et al.*, 2015).

Peroxidases PRX33 and PRX34 have also been shown to act in salicylic acid (SA) signalling, as *asFBP1.1* plants are impaired in the expression of several SA‐responsive genes and in SA‐induced NPR1 monomerization (Mammarella *et al.*, 2015). The formation of NPR1 monomers is dependent on the cellular redox state and is also a crucial component of the SA‐mediated signalling pathway (Mou *et al.*, 2003; Tada *et al.*, 2008). Two studies on the cell wall proteome of *Arabidopsis* leaves also drew attention to PRX33 or PRX34 in responses to pathogen infection and redox imbalances. PRX34 is over‐represented in the apoplastic proteome of *Verticillium longisporum*‐infected leaves (Floerl *et al.*, 2012), and PRX33 and PRX34 are both more abundant in cell wall protein extracts of ascorbate‐deficient *Arabidopsis* leaves relative to those of wild‐type plants (Sultana *et al.*, 2015).

In addition to NADPH oxidases and apoplastic peroxidases, AOs and PAOs have been proposed as alternative sources of extracellular ROS accumulation. Polyamines are catabolized by copper‐containing AOs (CuAOs) and FAD‐dependent PAOs. PAOs catalyse the oxidation of spermine (Spm), spermidine (Spd) and/or their acetylated derivatives at the secondary amino groups (Angelini *et al.*, 2010). In *Arabidopsis*, five *PAO* isoforms (*AtPAO1*–*AtPAO5*) have been identified, and the main physiological role of these proteins has been linked to compartment‐specific H_2_O_2_ synthesis in different phases of development and differentiation, as well as in the course of defence mechanisms against pathogens and abiotic stress (Andronis *et al.*, 2014; Fincato *et al.*, 2012; Takahashi *et al.*, 2010). *Arabidopsis* plants inoculated with an avirulent strain of *P. syringae* pv. *tomato* DC3000 (avrRpm1) accumulate polyamines, and infiltration of polyamines into the apoplast of leaves causes an oxidative burst and subsequent cell death (Yoda *et al.*, 2009).

The *Arabidopsis thaliana*–*Alternaria brassicicola* pathosystem has been widely used to study plant defence and fungal pathogenesis strategies (Schenk *et al.*, 2003; Su’udi *et al.*, 2011). We have reported previously that the functional* Arabidopsis* NADPH oxidase RBOHD (respiratory burst oxidase homologue D) is required for an extracellular oxidative burst when plants are spray inoculated with a conidial suspension of the necrotrophic fungus *A. brassicicola*. An *rbohd* knockout line showed reduced susceptibility to the pathogen and increased spread of cell death, indicating that RBOHD contributes to the success of the colonization process and is also engaged in the regulation of *A. brassicicola*‐induced host cell death. Finally, an interplay between RBOHD, ethylene and SA was demonstrated, and diverse roles of RBOHD‐dependent ROS were suggested between cells that were affected directly by the fungus and cells that were in neighbouring positions (Pogány *et al.*, 2009).

The necrotrophic fungus *A. brassicicola* causes black spot disease and is an economically important pathogen of Brassicaceae species (Thomma, 2003). The interaction between *A. brassicicola *strain MUCL 20297 and *Arabidopsis thaliana* ecotype Col‐0 (both used throughout this work) is considered to be incompatible (Narusaka *et al.*, 2005; Thomma *et al.*, 1998). Nevertheless, our experimental conditions (which were excessively favourable for the fungus) enabled considerable pathogen growth (Pogány *et al.*, 2009).

The various enzymatic sources of extracellular ROS in diseased (or wounded) plants are typically examined separately and in different pathosystems. Sometimes, when these sources are investigated together, enzyme inhibitors are used to distinguish between NADPH oxidase‐, class III peroxidase‐ or PAO‐derived ROS (Dmochowska‐Boguta *et al.*, 2013; O’Brien *et al.*, 2012; Roach *et al.*, 2015). As an extension of our work on the cellular functions of the NADPH oxidase RBOHD (Pogány *et al.*, 2009), in this study, we provide results on the contribution of peroxidases PRX33 and PRX34 to a pathogen‐induced apoplastic oxidative burst. The experiments are performed with the same *Arabidopsis*–*Alternaria* pathosystem as in the RBOHD study, using knockout and knockdown insertion mutant and gene‐silenced plant samples. Some likely candidates of the RBOHD, PRX33 and PRX34 signalling networks are also exposed.

## Results

### Elicitation of *Arabidopsis *leaf cells by *A. brassicicola *activates *PRX33 *and *PRX34* transcription

**Figure 1:**
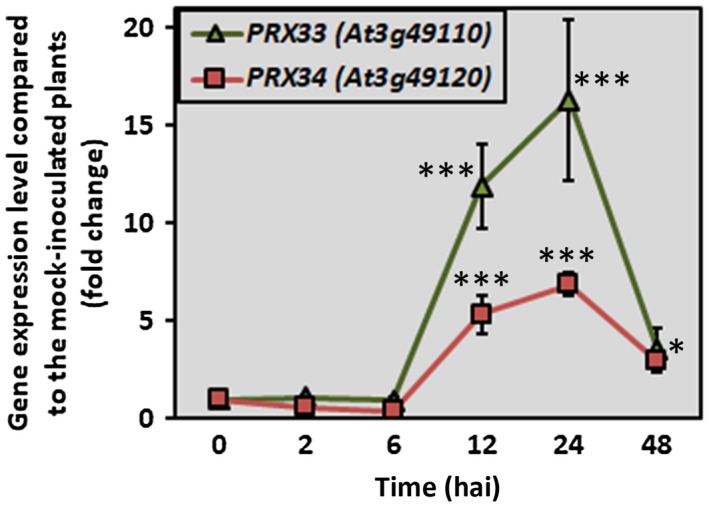
*Alternaria brassicicola*‐induced activation of *PRX33* and *PRX34* genes in wild‐type *Arabidopsis* plants. Five‐ to six‐week‐old *Arabidopsis* plants (whole rosettes) were spray inoculated with *A. brassicicola* conidial suspension at a concentration of 5 × 10^5^ conidia/mL distilled water. Data represent the mean of three independent biological samples with three technical replicates each. Statistical analysis was performed using Mann–Whitney *U*‐test. Asterisks indicate statistically significant differences (*α = 0.05, ***α = 0.001).

To explore the contribution of apoplastic peroxidases and PAOs in the oxidative burst, we monitored mRNA abundance for peroxidase genes *PRX33* and *PRX34* and PAO isoforms *PAO1*, *PAO2*, *PAO3*, *PAO4* and *PAO5* in our pathosystem. Expression of the two cell wall peroxidases was clearly induced after 12 h, and the highest expression levels were observed at 24 h after inoculation (hai) for both *PRX* genes in comparison with mock‐inoculated plants (Fig. 1). In contrast, transcription of *PAO1*, *PAO2*, *PAO3* and *PAO4* remained unaffected and *PAO5* mRNA levels were repressed by elicitation with *A. brassicicola* at 24 hai, indicating that they are not involved in the *A. brassicicola*‐induced oxidative burst and other host responses (Fig. S1, see Supporting Information).

### Knockdown of *PRX33 *and *PRX34 *transcript levels reduces symptoms and colonization success of *A. brassicicola*

In a process to select *Arabidopsis* genotypes with adequate *PRX33* and *PRX34* knockdown mRNA levels, T‐DNA insertion mutants *prx33* (SALK_062314C) and *prx34* (SALK_051769C) (Passardi *et al.*, 2006), together with the line *asFBP1.1* expressing a French bean class III peroxidase in an antisense orientation (Bindschedler *et al.*, 2006), were initially included in this work. Transcript levels of peroxidase gene *PRX33* were significantly reduced in the *prx33* insertion line and in *asFBP1.1*, but mRNA levels for the peroxidase gene *PRX34* were not sufficiently suppressed in our hands in the *prx34* insertion line or in the *asFBP1.1* line. Therefore, we created a *Tobacco rattle virus* (TRV) vector‐based virus‐induced gene silencing (VIGS) construct (Hayward *et al*., 2011) that provided *Arabidopsis* plants with markedly reduced *PRX34* (and *PRX33*) transcript levels. This *TRV‐GFP‐PRX* VIGS construct is hereafter abbreviated as *TRV‐PRX*. Diminished activity of *PRX33* and *PRX34* genes in the *prx33* insertion line and in plants treated with the *TRV‐PRX* VIGS construct is shown in Figs S2 and S3 (see Supporting Information). All subsequent pathogen and ROS detection assays aimed at the characterization of extracellular PRX33 and PRX34 functions in *Arabidopsis* during fungal pathogenesis were conducted with the *prx33* (SALK_062314C) line and with plants carrying the *TRV‐PRX* VIGS construct. A previously characterized *rbohd* NADPH oxidase knockout line (SALK_070610C) was also included in some experiments (Pogány *et al.*, 2009).

**Figure 2:**
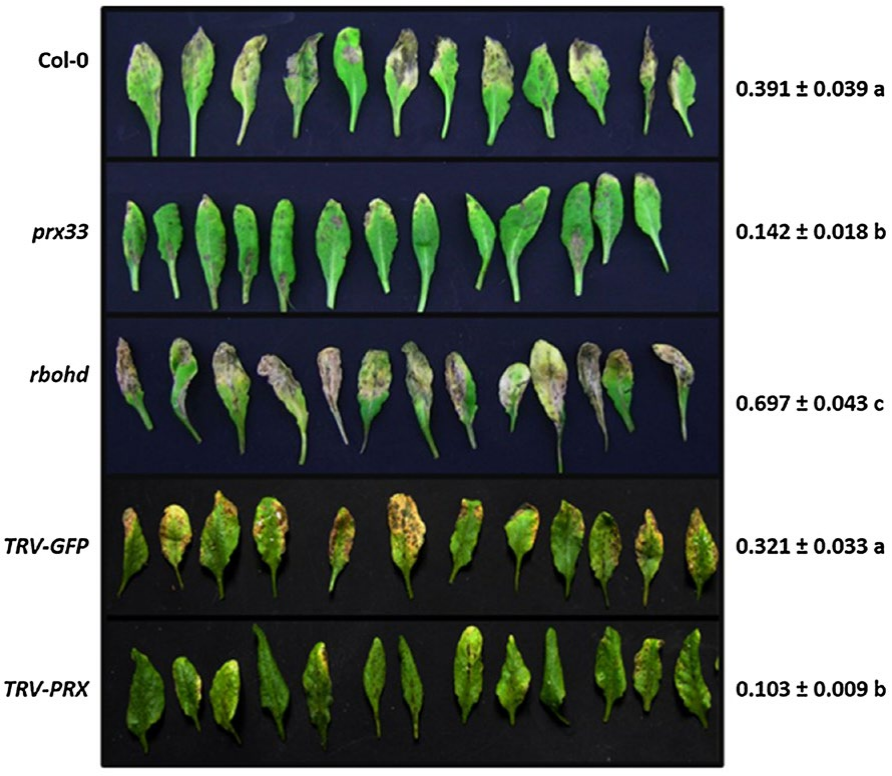
Symptoms of *Alternaria brassicicola* infection are suppressed in *Arabidopsis* plants with reduced *PRX33*/*PRX34* mRNA levels. Leaves of *A. brassicicola*‐infected Col‐0, *prx33* knockdown mutant, *rbohd* knockout mutant, *TRV‐GFP* and *TRV‐PRX* plants are shown 10 days after inoculation. Five‐ to six‐week‐old *Arabidopsis* plants (whole rosettes) were spray inoculated with *A. brassicicola* conidial suspension at a concentration of 5 × 10^5^ conidia/mL distilled water. Detached leaves in middle positions (leaf levels 5–8) were evaluated. Results are presented as the chlorotic and necrotic leaf area compared with the total surface area of leaf blades analysed by ImageJ (1 is equal to 100% leaf surface), and represent the means of two experiments (*n* = 30 for each genotype/treatment) ± standard error (SE). Different letters indicate statistically significant differences between genotypes/treatments using Tukey’s *post hoc* test. Increased symptoms of *A. brassicicola* on leaves of the *rbohd* mutant are included as a reference.

**Figure 3:**
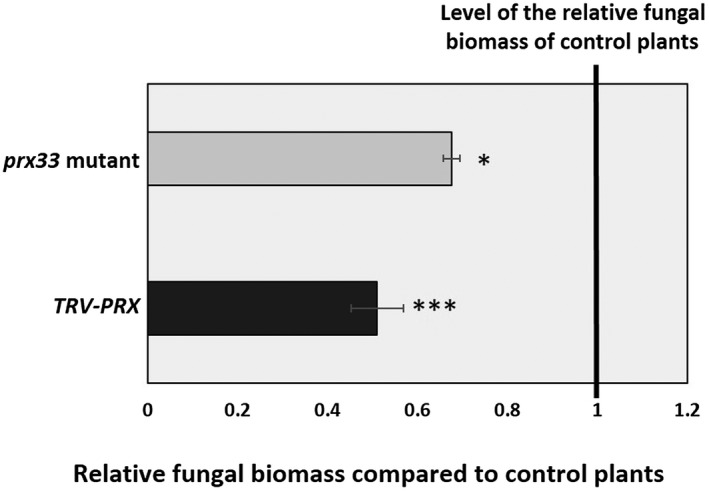
*Alternaria brassicicola* biomass in *prx33 *T‐DNA insertion line and in gene‐silenced (*TRV‐PRX*) plants. Five‐ to six‐week‐old *Arabidopsis* plants (whole rosettes) were spray inoculated with *A. brassicicola* conidial suspension at a concentration of 5 × 10^5^ conidia/mL distilled water. The results show the average of two experiments, each comprising three biological samples (each sample composed of a pool of three *Arabidopsis* rosettes), analysed in three technical replicates. Statistical analysis was performed using Student’s *t*‐test. Asterisks indicate statistically significant differences between *PRX* knockdown *Arabidopsis* plants (*prx33*, *TRV‐PRX*) and their controls (Col‐0, *TRV‐GFP*) at 10 days after inoculation with *A. brassicicola* (*α = 0.05, ***α = 0.001).

Down‐regulation of *PRX33* and *PRX34* yielded reduced fungal symptoms on *Arabidopsis* leaves. The percentage of leaf chlorosis and necrosis triggered by *A. brassicicola* in plants with diminished *PRX33* and *PRX34* transcript levels was only 32%–36% of that in plants showing wild‐type *PRX33/PRX34* transcript levels (Fig. 2). The fungal biomass of *A. brassicicola* was also lower in *prx33*/*prx34* knockdown plants, representing 68% (*prx33*) and 51% (*TRV‐PRX*) of that of corresponding inoculated control plants with wild‐type *PRX33/PRX34* transcript levels (Fig. 3).

### H_2_O_2_ accumulation

H_2_O_2_ was detected by a non‐fluorescent 3,3′‐diaminobenzidine (DAB) histochemical staining procedure, which is based on an H_2_O_2_‐dependent peroxidase‐catalysed polymerization reaction (Thordal‐Christensen *et al.*, 1997), and a fluorescent 2′,7′‐dichlorofluorescein diacetate (DCFH‐DA) method. DCFH‐DA also reacts with H_2_O_2_ in the presence of peroxidases, yielding a fluorescent DCF product (Bozsó *et al.*, 2005). Inoculated leaves were observed microscopically for the DAB‐stained samples and visualized with UV light for the DCFH‐DA‐stained leaves, where the intensity of the fluorescence signal released by individual leaves was detected.

**Figure 4:**
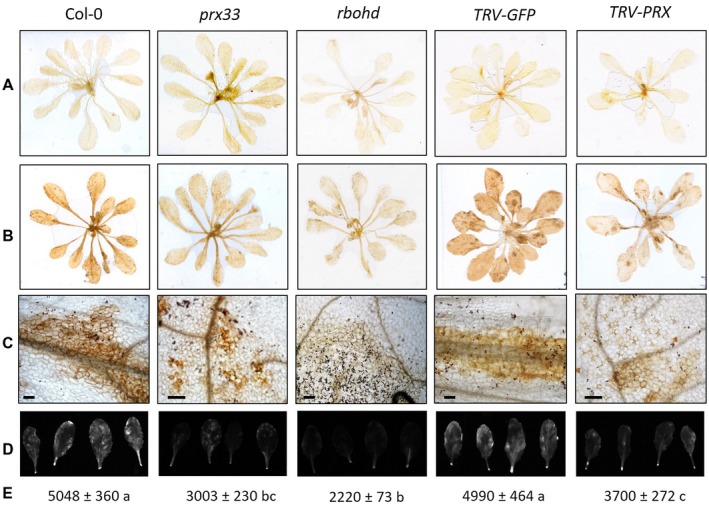
Hydrogen peroxide (H_2_O_2_) production in *Arabidopsis* plants with reduced cell wall peroxidase and NADPH oxidase activity. The accumulation of H_2_O_2_ was detected in wild‐type (Col‐0), *prx33* and *rbohd* T‐DNA insertion lines and in *TRV‐GFP* and *TRV‐PRX* gene‐silenced *Arabidopsis* plants by 3,3′‐diaminobenzidine (DAB) (A–C) and 2′,7′‐dichlorofluorescein diacetate (DCFH‐DA) (D, E) staining methods. Five‐ to six‐week‐old *Arabidopsis* plants (whole rosettes) were spray inoculated with *Alternaria brassicicola* conidial suspension at a concentration of 5 × 10^5^ conidia/mL distilled water. The stainings were carried out with mock‐inoculated (A) and *Alternaria brassicicola*‐infected (B–E) plants (2 days after inoculation). Bars, 50 µm. The intensity of the fluorescence signal emitted by DCFH‐DA‐stained leaves (D) was quantified under UV light using an AlphaImager Mini gel documentation system (E). Results are presented as an average pixel fluorescence intensity and represent the means of two experiments (*n* = 30 leaves in the middle position for each genotype/treatment) ± standard error (SE). Statistical analysis was performed using one‐way analysis of variance (ANOVA) and Tukey’s *post hoc* test. Different letters indicate statistically significant results. *prx33* and *TRV‐PRX* plants accumulate less H_2_O_2_ than the corresponding control plants. Microscopic observation of the patterns of H_2_O_2_ accumulation reveals that *prx33* and *TRV‐PRX *plants show apoplastic H_2_O_2_ accumulation in a reduced number of cells in comparison with the corresponding control plants after inoculation with *A. brassicicola*. Leaves of the *rbohd* knockout line lacking apoplastic H_2_O_2_ accumulation after *A. brassicicola* infection are shown as a reference. Decreased H_2_O_2_ production in *rbohd* plants was also confirmed by the DCFH‐DA staining method. The mean level of base fluorescence for mock‐inoculated Col‐0 plants was 1240 ± 34.

Knockdown of *PRX33* and *PRX34* transcript levels led to decreased H_2_O_2_ formation in *Arabidopsis* leaves challenged with *A. brassicicola* (Fig. 4). This altered response was quantitative because the apoplastic burst was not completely abolished, but the number of cells exhibiting a burst of extracellular H_2_O_2_ was reduced (Fig. 4C). This is in contrast with the fully inhibited apoplastic ROS accumulation in the *rbohd* mutant (Pogány *et al.*, 2009). We quantified the number of cells showing extracellular H_2_O_2_ accumulation at 2 days after inoculation with *A. brassicicola* for 20 randomly selected infection sites, observing leaves from two independent experiments. This evaluation revealed that infection sites in wild‐type (Col‐0) plants were composed of an average of 42.6 ± 5.7 cells showing ROS accumulation relative to 16.0 ± 2.5 cells in *prx33* plants. Likewise, infection sites in *TRV‐GFP* plants with normal *PRX33/PRX34* transcript levels comprised an average of 34.4 ± 3.8 cells exhibiting H_2_O_2_ accumulation relative to 17.0 ± 1.2 cells in *prx33/prx34* under‐producing *TRV‐PRX* plants.

The adaptation of a quantifiable, fluorescent, ROS detection method suitable for whole leaves gave us similar results on the accumulation of ROS in our *A. brassicicola*‐infected genotypes/treatments, confirming the lower levels of ROS in *prx33*/*prx34* under‐producing *Arabidopsis* plants (Fig. 4D). ROS formation, represented by the intensity of fluorescence signals, in leaves of infected *prx33* plants was 59% of that in leaves of the corresponding infected wild‐type plants, and in leaves of infected *TRV‐PRX* plants was 74% of that in leaves of infected *TRV‐GFP* plants, measured 2 days after inoculation with *A. brassicicola*. The fluorescence signal intensity in leaves of NADPH oxidase mutant *rbohd* plants was also detected as a reference, and a marked reduction in ROS accumulation was observed. In *rbohd* plants, the ROS‐related fluorescence signal intensity was 44% of that of wild‐type Col‐0 plants.

### Leaf senescence clearly affects *RbohD*, *PRX33 *and *PRX34* mRNA levels

**Figure 5:**
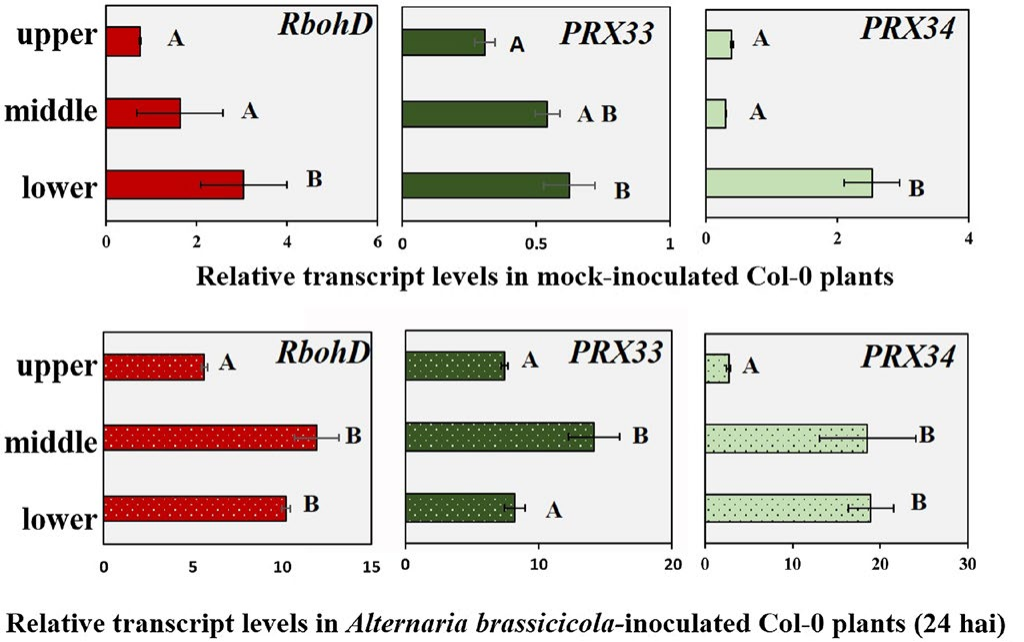
Transcript abundance of *RbohD*, *PRX33* and *PRX34* genes at three leaf levels. Gene expression was analysed in leaves representing three consecutive physiological states (upper, middle and lower). Five‐ to six‐week‐old wild‐type *Arabidopsis* plants (whole rosettes) were spray inoculated with *Alternaria brassicicola* conidial suspension at a concentration of 5 × 10^5^ conidia/mL distilled water. Mock‐inoculated and *A. brassicicola*‐infected [24 h after inoculation (hai)] plants were assayed. Relative gene expression was calculated using the comparative 2^–ΔΔCT^ method and *At4g26410* as a reference gene. The results show the average of two experiments, each comprising three biological samples (each sample composed of a pool of the corresponding leaves of three *Arabidopsis* rosettes), analysed in three technical replicates. True leaves growing on one plant were divided evenly into three age groups: lower leaves (typically in positions 1–4), middle leaves (in positions 5–8) and upper leaves (in positions 9–12). Different letters represent relative transcript values significantly different at *P* < 0.05. Statistical analysis was performed using one‐way analysis of variance (ANOVA) and Tukey’s *post hoc* test.

The results of several studies have indicated functional interactions between the *Arabidopsis* NADPH oxidase RBOHD and the senescence hormone ethylene or its precursor 1‐aminocyclopropane‐1‐carboxylic acid (ACC) (Bouchez *et al.*, 2007; Pogány *et al*., 2009; Mersmann *et al.*, 2010; Yao *et al.*, 2017). This observation prompted us to investigate the effect of leaf age on the activity of genes (*RbohD*, *PRX33* and *PRX34*) encoding crucial ROS‐generating proteins in *Arabidopsis*, before and after elicitation with *A. brassicicola*. Sampling was performed by dividing true leaves growing on one plant evenly into three age groups: oldest leaves (in leaf positions 1–4), middle leaves (in leaf positions 5–8) and youngest leaves (in leaf positions 9–12). The basal level of transcript abundance examined in mock‐inoculated wild‐type plants was highest in the oldest bottom leaves for all three ROS‐producing proteins, and non‐senescent or younger leaves exhibited moderate or lower basal transcript abundance for *RbohD*, *PRX33* and *PRX34* genes (Fig. 5). Twenty‐four hours after inoculation with *A. brassicicola*, however, non‐senescent middle leaves responded to fungal infection with the highest induction of *RbohD*, *PRX33* and *PRX34* transcript levels (Fig. 5).

### Searching for new components in the PRX33/PRX34 and RBOHD signalling networks

**Figure 6:**
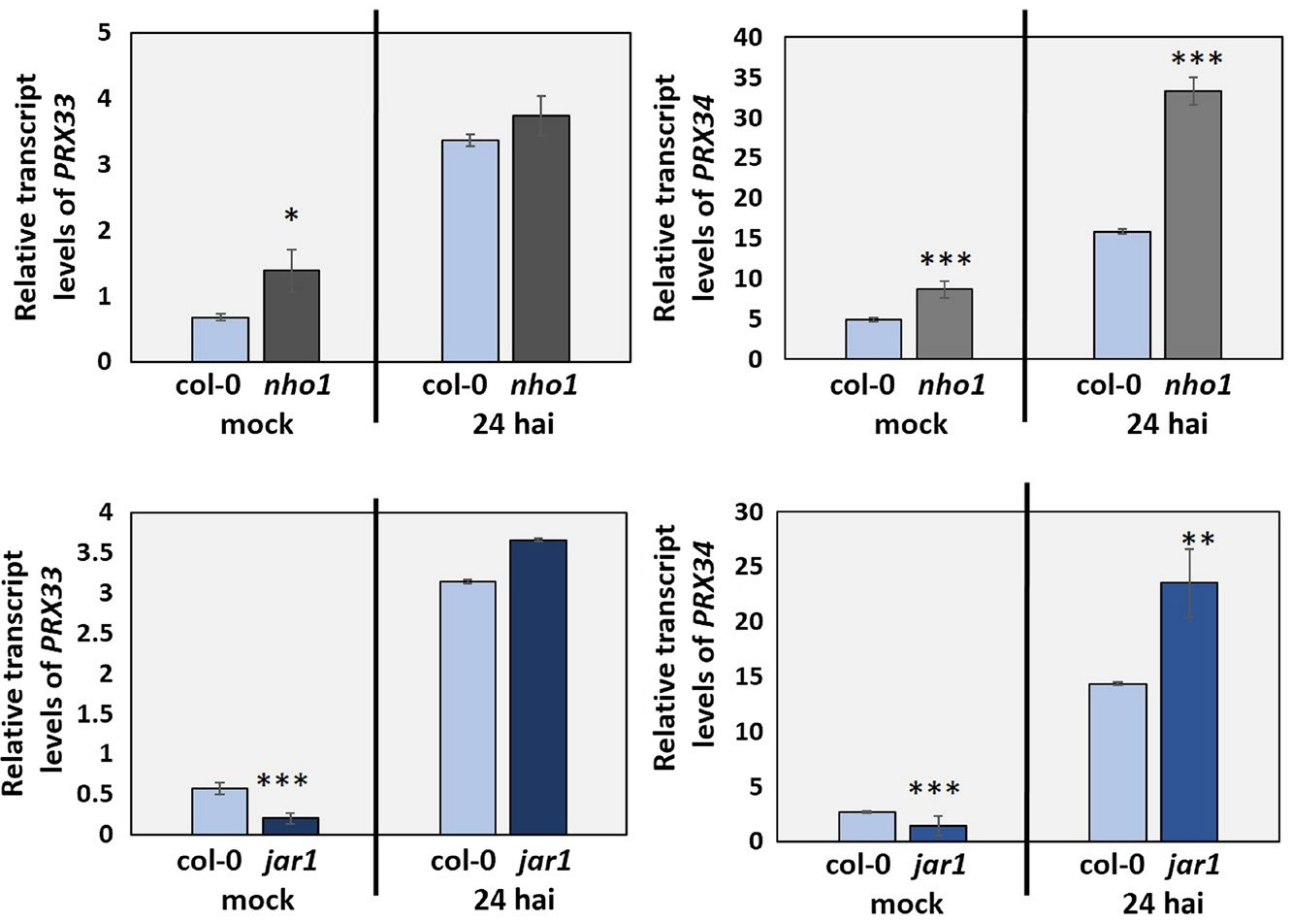
Transcript levels of *PRX33* and *PRX34* genes in *nho1* and *jar1* T‐DNA insertion lines relative to wild‐type plants. The level of gene expression was analysed before and after inoculation with *Alternaria brassicicola* [24 h after inoculation (hai)]. Dysfunctions in NHO1 and JAR1 activity affect *PRX33* and *PRX34* mRNA levels. Five‐ to six‐week‐old *Arabidopsis* plants (whole rosettes) were spray inoculated with *A. brassicicola* conidial suspension at a concentration of 5 × 10^5^ conidia/mL distilled water. The results show the average of two experiments, each comprising three biological samples (each sample composed of a pool of three *Arabidopsis* rosettes), analysed in three technical replicates. Statistical analysis was performed using Student’s *t*‐test. Asterisks indicate statistically significant differences (*α = 0.05, **α = 0.01, ***α = 0.001).

In a pursuit to find unknown elements of the RBOHD or PRX33/PRX34 cellular interaction networks, two approaches were employed. First, T‐DNA insertion lines with perturbed activity of key *Arabidopsis* immune regulators were inoculated with *A. brassicicola*. Mutants corresponding to seven crucial determinants [BIK1, NPR1, MPK6, NIA2, EIN2, JAR1 (JASMONATE RESISTANT1) and NHO1 (NON‐HOST RESISTANCE TO P. SYRINGAE PV. PHASEOLICOLA 1)] showed altered host responses (in comparison with wild‐type plants) after inoculation with *A. brassicicola*. Six were further investigated. BIK1 was excluded because its role in the regulation of RBOHD activity has been confirmed (Kadota *et al.*, 2014; Li *et al.*, 2014). Mutants affected in the activity of two of these pivotal plant immunity factors, JAR1 and NHO1, exhibited consistently altered *PRX33* and *PRX34* transcript levels (Fig. 6), but the other four mutants showed wild‐type transcript levels for *PRX33* and *PRX34* genes. Interestingly, no apparent differences in *RbohD* mRNA levels could be detected between any of the immune mutants and the wild‐type, before or after inoculation with *A. brassicicola*.

**Figure 7:**
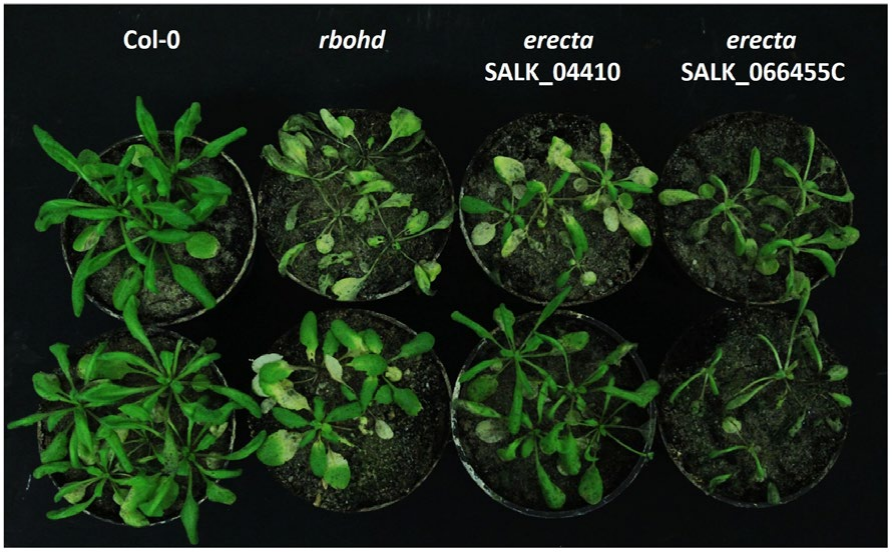
*Alternaria brassicicola*‐infected Col‐0, *rbohd*, *erecta* SALK_04410 and *erecta* SALK_066455C mutant *Arabidopsis* plants. Fungal symptoms were photographed at 7 days after inoculation. T‐DNA insertion lines with mutations in the genomic sequence of *RbohD* (*At5g47910*) and *ERECTA* (*At2g26330*) genes were used. Necrotic fungal symptoms are more intense in the two *erecta* mutants, as in the *rbohd* mutant, in comparison with the Col‐0 wild‐type. Five‐ to six‐week‐old *Arabidopsis* plants (whole rosettes) were spray inoculated with *A. brassicicola* conidial suspension at a concentration of 5 × 10^5^ conidia/mL distilled water.

**Figure 8:**
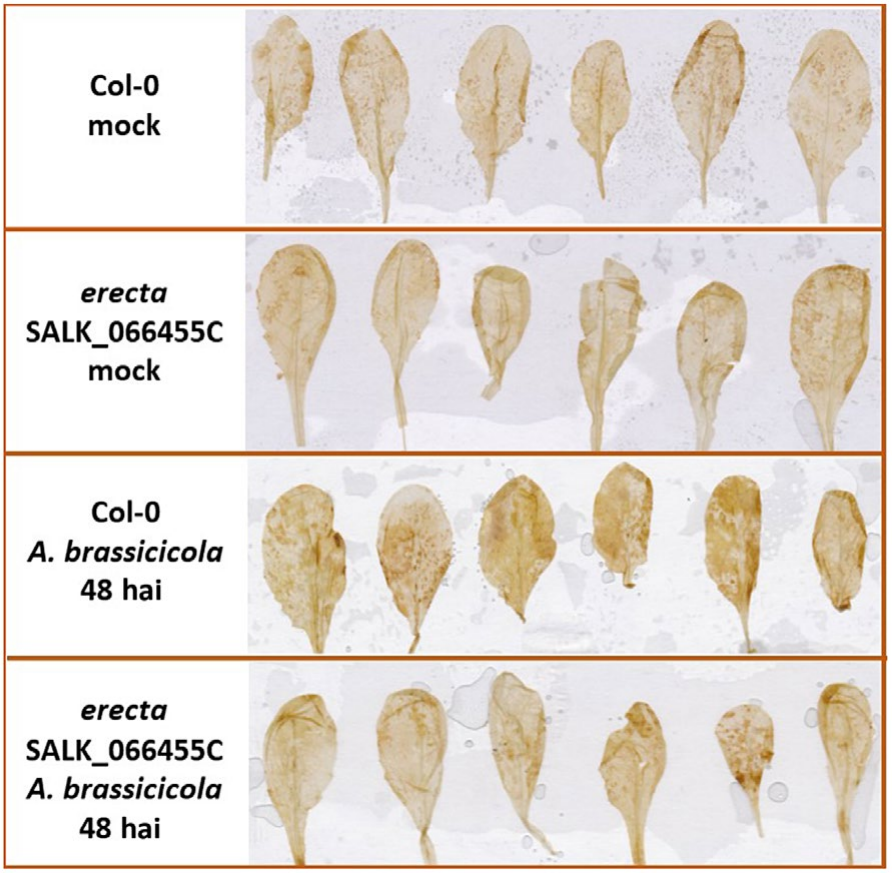
Hydrogen peroxide (H_2_O_2_) production in wild‐type (Col‐0) and *erecta* T‐DNA insertion line *Arabidopsis* leaves. The accumulation of H_2_O_2_ was visualized by 3,3′‐diaminobenzidine (DAB) staining. Five‐ to six‐week‐old *Arabidopsis* plants (whole rosettes) were spray inoculated with *Alternaria brassicicola* conidial suspension at a concentration of 5 × 10^5^ conidia/mL distilled water. The staining was carried out with mock‐inoculated and *A. brassicicola*‐infected plants [48 h after inoculation (hai)]. Leaves of the *A. brassicicola*‐infected *erecta* mutant show markedly reduced accumulation of H_2_O_2_. The detection was repeated twice using 15 plants for each treatment. Representative leaves are shown.

In a second approach, T‐DNA insertion lines affected in the activity of 11 selected target proteins were analysed. Earlier, these proteins had been reported to establish a physical interaction with RBOHD (Geisler‐Lee *et al.*, 2007; Jones *et al.*, 2014). Mutants with associated insertions in the following genes were included: *UDP‐Galactose Transporter 3*, *Annexin 1*, *Thioredoxin H‐type 7*, *Calmodulin 4*, *Glutathione S‐Transferase TAU 19*, *Quantitative Resistance to Plectosphaerella 1*, *Cell Elongation Protein*, *ADP‐Ribosylation Factor C1*, *Membrane‐Associated Progesterone Binding Protein 3*, *At4g37445 *without functional prediction and *NDR1/HIN1‐like 3 *(Table S1, see Supporting Information). Two mutants in which the insertions could be linked to the same locus, *At2g26330* encoding *ERECTA* (*Quantitative Resistance to Plectosphaerella 1*), gave spreading cell death phenotypes on inoculation with *A. brassicicola*, similar to those seen on the *rbohd* mutant (Fig. 7). The disease responses of the mutants representing the other 10 RBOHD interactors were the same as in the wild‐type. One of the *erecta* T‐DNA insertion lines, SALK_066455C, was also assayed for ROS formation after inoculation with *A. brassicicola*. The accumulation of H_2_O_2_, visualized by DAB staining, was highly compromised in whole leaves of the *erecta* mutant at 48 hai with *A. brassicicola* (Fig. 8).

## Discussion

### *Alternaria brassicicola* infection activates *PRX33* and *PRX34* genes

The mRNA expression of genes encoding ROS‐producing proteins was examined in *A. brassicicola*‐infected *Arabidopsis *plants. *PRX33* and *PRX34* mRNA levels were elevated even at 12 hai, reaching their climax at 24 hai. The analysis of NADPH oxidase *RbohD* transcript levels, investigated in the same pathosystem, revealed a similar transcriptional response (Pogány *et al.*, 2009). The induction of *PRX34* gene expression was also reported in *Sclerotinia sclerotiorum*‐infected *Brassica napus* plants at 12–48 hai (Yang *et al.*, 2007). *PAO1*, *PAO2*, *PAO3*, *PAO4* and *PAO5* encoding genes, however, did not show *A. brassicicola*‐induced activation at 24 hai with the fungus (Figs 1 and S1).

These data suggest that the apoplastic peroxidases PRX33 and PRX34 play important roles in the response of *Arabidopsis* to *A. brassicicola*, but the significance of PAO1, PAO2, PAO3, PAO4 and PAO5 was not proven.

### PRX33 and PRX34 contribute markedly to the *A. brassicicola*‐induced accumulation of H_2_O_2_

Impaired RBOHD function leads to the abolishment of apoplastic ROS accumulation in *Alternaria*‐infected *Arabidopsis* cells, whereas down‐regulation of PRX33/PRX34 activity results in a reduced number of leaf cells exhibiting the burst of extracellular ROS (Fig. 4). These results reveal that the RBOHD and PRX33/PRX34 ROS‐generating systems contribute simultaneously to the *Alternaria*‐induced extracellular oxidative burst in *Arabidopsis*. The reduced number of leaf cells showing H_2_O_2_ accumulation indicates a role of the apoplastic peroxidases PRX33/PRX34 in the spread of intercellular ROS signalling initiated by RBOHD (Gilroy *et al.*, 2014, 2016).

Some crucial *Arabidopsis* stomatal responses mediated by the plant hormone cytokinin have been recently linked to the activity of peroxidases PRX33 and PRX34, together with certain other apoplastic peroxidases (Arnaud *et al.*, 2017). It was shown that cytokinin‐induced stomatal closure, ROS accumulation in guard cells and stomatal immunity to coronatine‐deficient *P. syringae* pv. *tomato* DC3000 bacteria were dependent on the activity of the extracellular peroxidases PRX33 and PRX34.

### Necrotic symptoms of the fungal infection are enhanced by PRX33/PRX34‐derived ROS

Down‐regulation of PRX33 and PRX34 led to reduced necrotic fungal symptoms on *Arabidopsis* leaves (Fig. 2) and the relative fungal biomass of *A. brassicicola* was lower in *prx33*/*prx34* knockdown plants (Fig. 3). These results suggest that, similar to RBOHD, functional PRX33 and PRX34 and their ROS products support the growth of the fungus. However, PRX33/PRX34 activity leads to increased tissue necrosis, in contrast with functional RBOHD, which suppresses tissue necrosis.

It is unclear whether the reduced necrotic symptoms in our *prx33/prx34* under‐producers are the result of partially compromised cell death induction or simply the consequence of limited necrotrophic pathogen growth. Mammarella *et al. *(2015) reported that *asFBP1.1* *prx* knockdown plants retained the ability to mount a hypersensitive cell death response to attacks by avirulent *P. syringae* strains, suggesting that the apoplastic peroxidases PRX33/PRX34 are dispensable in pathogen‐induced cell death in *Arabidopsis*.

The inhibition of RBOHD activity in a T‐DNA mutant resulted in more intense tissue necrosis (increased spread of cell death) after inoculation with *A. brassicicola*. Fungal colonization, however, was suppressed in *rbohd* knockout plants relative to the wild‐type. Thus, RBOHD regulates host cell death on *A. brassicicola* infection and functional RBOHD supports the colonization of *Arabidopsis* by this necrotrophic fungus (Pogány *et al.*, 2009).

### Leaf senescence modulates the transcript levels of ROS‐producing enzymes

Leaf senescence has been associated with elevated ROS levels and reduced antioxidant capacity (Barna *et al.*, 2012). In our studies, senescence was connected with an increased basal transcript level of pivotal ROS‐producing enzymes, but also with a reduced ability to develop a full transcriptional response of *RbohD*, *PRX33* and *PRX34* genes following the attack of a necrotrophic fungus (Fig. 5).

This is in accord with our microscopic observations, where cells in older leaves of *Arabidopsis* plants exhibited a mild, but uniform, induction of apoplastic basal H_2_O_2_ accumulation, but younger (non‐senescent) leaves responded to *A. brassicicola* infection with a more pronounced accumulation of extracellular H_2_O_2_. Characteristic spatio‐temporal mRNA expression patterns of NADPH oxidase isoforms *RbohD* and *RbohF* have been reported recently in unchallenged and pathogen‐elicited *Arabidopsis* plants (Morales *et al.*, 2016).

### Quest for new PRX33/34 and RBOHD interactors

In a search for new components in the PRX33/PRX34 and RBOHD signalling networks, numerous Arabidopsis mutants were tested.

T‐DNA insertion lines *jar1* and *nho1* consistently exhibited altered *PRX33/PRX34* transcript abundance, both before and after inoculation with *A. brassicicola* (Fig. 6). JAR1 encodes a jasmonic acid‐amido synthetase which catalyses the formation of a biologically active jasmonyl‐isoleucine (JA‐Ile) conjugate. This amino acid is necessary for optimal signalling in some jasmonate responses in *Arabidopsis* (Staswick and Tiryaki, 2004; Staswick *et al.*, 2002). Synthesis of the JA‐Ile conjugate by JAR1 and related JA‐conjugating enzymes is required for the plant immune system (Ryu *et al.*, 2004; Staswick *et al.*, 1998) and for various abiotic stress responses (Rao *et al.*, 2000). Functional JAR1 alleviates the oxidative burst in *Arabidopsis* seedlings triggered by the bacterial MAMP elicitor flagellin (Yi *et al.*, 2014). An interaction between JAR1 and ROS has been reported during cell wall damage‐induced lignin synthesis in *Arabidopsis* (Denness *et al.*, 2011).

NHO1 protein is a glycerol kinase, which converts glycerol to glycerol 3‐phosphate and performs a rate‐limiting step in glycerol metabolism (Kang *et al.*, 2003). This protein is also known as GLI1 or GLYCEROL‐INSENSITIVE 1 (Eastmond, 2004). *Arabidopsis* *NHO1* transcript levels are induced by non‐host *Pseudomonas* bacterial strains, and functional NHO1 is required for resistance against non‐host and avirulent *Pseudomonas* strains, as well as against the necrotrophic fungal pathogen *Botrytis cinerea*. However, the *NHO1* gene is ineffective against virulent *P. syringae* pv. *tomato* DC3000, and its transcription is suppressed by the DC3000 strain. Interestingly, the JA signalling pathway is specifically required for *NHO1* suppression by DC3000 bacteria (Kang *et al.*, 2003; Li *et al.*, 2005; Lu *et al.*, 2001). Although the fact that NHO1 is a significant component of the immune system of *Arabidopsis* is well established, its specific functions in immune signalling are still largely elusive (Lu *et al.*, 2010; Maeda *et al.*, 2010). Nonetheless, a connection between ROS production and NHO1 activity is emerging (Wang *et al.*, 2014). Perturbation of the cellular redox status in plants, triggered by catalase or glycolate oxidase deficiency or by treatment with the ROS‐generating compound methylviologen, significantly alters the transcript level of *NHO1* (Chaouch *et al.*, 2010; El‐Maarouf‐Bouteau *et al.*, 2015; Rojas *et al.*, 2012). Conversely, plants with disturbed glycerol metabolism or with impaired *NHO1* gene expression have been shown to exhibit elevated H_2_O_2_ formation (Hu *et al.*, 2014; Li *et al.*, 2016). Our results, revealing enhanced *PRX33/PRX34* gene activity in the *nho1* knockout (in comparison with wild‐type plants), before or after inoculation with *A. brassicicola*, indicate that these extracellular peroxidases may be partly responsible for the observed interaction between ROS and NHO1 (Fig. 6).

In a different set of experiments, *Arabidopsis* plants carrying mutations in the *ERECTA* gene were shown to display spreading necrosis symptoms on *A. brassicicola* infection, very similar to those observed on the *rbohd* mutant (Fig. 7). Inoculated leaves of the *erecta* mutant (SALK_066455C) also exhibited reduced H_2_O_2_ accumulation (Fig. 8). ERECTA has been reported previously to establish a physical interaction with RBOHD (Geisler‐Lee *et al.*, 2007; Jones *et al.*, 2014).

ERECTA (QUANTITATIVE RESISTANCE TO PLECTOSPHAERELLA 1, AGI locus code: *At2g26330*) has been connected to thermotolerance. It belongs to the protein kinase superfamily and to the serine/threonine (Ser/Thr) protein kinase family, which includes proteins that are associated with development, pathogen defence and phytohormone perception (Sánchez‐Rodríguez *et al*., 2009; Torii *et al*., 1996). ERECTA plays a role in the immune response of *Arabidopsis* because *erecta* mutant plants are more susceptible than wild‐type plants to a variety of pathogens (*Magnaporthe oryzae*, *Plectosphaerella cucumerina*, *Verticillium longisporum*) (Haffner *et al.*, 2014; Llorente *et al.*, 2005; Takahashi *et al.*, 2016). *Fusarium graminearum* infection apparently down‐regulates mRNA abundance for the *ERECTA* gene in *Arabidopsis* pistils (Mondragon‐Palomino *et al*., 2017). Results in our study demonstrate that ERECTA is also required for ROS accumulation and the suppression of necrotic symptoms caused by *A. brassicicola*, a necrotrophic fungal pathogen. ERECTA not only physically interacts with RBOHD (one of the major cellular sources of apoplastic ROS) in *Arabidopsis*, but has been shown to be part of a signalling pathway responsible for ROS sensing and redox‐mediated cortex proliferation in the roots (Cui *et al.*, 2014). Recently, the ROS responsivity of the *ERECTA *gene has been exposed in methylviologen‐treated *Arabidopsis* rosettes (Han *et al.*, 2014).

Taken together, we consider JAR1 and NHO1 to be potential members of the PRX33/PRX34 cellular signalling system and ERECTA to be an expected component of the RBOHD interaction network in *Arabidopsis* leaf cells when plants are challenged by the necrotrophic fungus *A. brassicicola*.

In this study, we have provided information on the simultaneous and comparable contribution of class III peroxidases PRX33 and PRX34 and NADPH oxidase RBOHD to the apoplastic oxidative burst investigated in the same *Arabidopsis*–*Alternaria* pathosystem. We have also presented microscopic images of the altered pattern of ROS accumulation in *Arabidopsis* plants with reduced PRX33 and PRX34 activity following inoculation with *A. brassicicola*. In contrast with the ROS‐producing NADPH oxidase RBOHD, which surprisingly inhibits the spread of cell death in pathogen‐infected *Arabidopsis* tissues (Pogány *et al.*, 2009), functional cell wall peroxidases PRX33 and PRX34 apparently enhance the development of necrotic symptoms triggered by infection with *A. brassicicola*. Fungal growth, however, is similarly stimulated by both RBOHD and the two apoplastic peroxidases. Finally, a senescence‐dependent distribution of *RbohD*, *PRX33* and *PRX34* transcripts is reported. As a conclusion of this study, ROS generated by the extracellular peroxidases PRX33 and PRX34 function in *Arabidopsis* as susceptibility factors when plants are challenged by the necrotrophic fungal pathogen *A. brassicicola*.

## Experimental Procedures

### Plant materials and growth conditions

Wild‐type (Col‐0), transgenic, mutant and gene‐silenced *Arabidopsis* plants were grown in a peat growing medium (Pindstrup Plus Orange, Pindstrup Mosebrug, Ryomgaard, Denmark) at 22 °C in a growth chamber under a 14‐h light/10‐h dark cycle with 80 µmol/m^2^/s irradiation. Seeds of the *Arabidopsis* line *asFBP1.1* expressing a French bean (*Phaseolus vulgaris*) peroxidase sequence in antisense orientation (Bindschedler *et al.*, 2006) were received from Professor F. M. Ausubel (Department of Genetics, Harvard Medical School, Boston, MA, USA). *Arabidopsis* T‐DNA insertion lines were ordered from the European *Arabidopsis* Stock Center. The list of *Arabidopsis *lines used in this study is provided in Table S1. Characterization of the *prx33* insertion line (SALK_062314C) is shown in Fig. S4 (see Supporting Information), whereas lines *prx34* (SALK 051769C) and *rbohd* (SALK_070610C) have been described previously (Passardi *et al.*, 2006; Pogány *et al.*, 2009). An *Arabidopsis* line expressing a green fluorescent protein coding mGFP‐ER sequence (Haseloff *et al.*, 1997), used for all VIGS experiments, was generated by a modified floral dip method (Logemann *et al.*, 2006).

### Cultivation of fungal strain and inoculation

*Alternaria brassicicola* strain MUCL20297, isolated from cabbage (Thomma *et al.*, 1998), was grown on potato dextrose agar medium at room temperature in the dark. The conidial suspension contained 5 × 10^5^ conidia/ml distilled water, and conidia were harvested from 1‐week‐old cultures. The inoculum was sprayed onto the leaf surface of 5–6‐week‐old *Arabidopsis* plants. The infected plants were incubated in 100% relative humidity in translucent plastic boxes under the same growing conditions as before. For mock‐inoculated plants, distilled water was sprayed onto the leaves and they were kept under the same conditions as the inoculated plants.

### Gene expression analysis

For total RNA extraction, *Arabidopsis* leaves were frozen in liquid nitrogen and ground with a mortar and pestle; 100 mg of plant material was used according to the manufacturer’s instructions (Total RNA Extraction Miniprep Kit, Viogene, Taipei, Taiwan). The RNA content and purity were analysed with a NanoDrop‐1000 (Thermo Fisher Scientific, Waltham, MA, USA) spectrophotometer.

Prior to cDNA synthesis all samples were subjected to DNase treatment (Invitrogen, Thermo Fisher Scientific, Waltham, MA, USA), DNA‐*free*™ DNA Removal Kit).

First‐strand cDNA was synthesized with a Thermo Scientific First Strand cDNA Synthesis Kit and used as a template for real‐time polymerase chain reaction (PCR) analysis in ten‐fold dilution. Real‐time PCRs were prepared using a Bioline (Taunton, MA, USA) SensiFAST SYBR^®^ No‐ROX Kit. The reactions were performed in a total volume of 15 μL containing 7.5 μL 2X Bioline SensiFAST qPCR Master Mix, 1.5–1.5 μL forward and reverse primers (10 μm), 2.5 μL template and 2 μL PCR‐grade water. The list of primer pairs used in this work is provided in Table S2 (see Supporting Information). All real‐time PCRs were carried out in a C1000 Touch Thermal Cycler equipped with a CFX96 Real‐Time PCR System (Bio‐Rad, Hercules, CA, USA). The following cycling conditions were selected: 95 °C for 3 min, then 40 cycles of 95 °C for 20 s, followed by 60 °C for 60 s. Finally, a melt curve analysis was performed to determine amplicon specificity with temperature increases from 65 to 95 °C in steps of 0.5 °C. Relative quantification analysis was performed using the comparative 2^−ΔΔCt^ method (Livak and Schmittgen, 2001). To evaluate the level of gene expression, the results were normalized using Ct values from the cDNA amplification of the constitutively expressed *Arabidopsis* *At4g26410* gene (Czechowski *et al.*, 2005).

### Determination of fungal biomass

Genomic DNA was isolated using the method of Brouwer *et al. *(2003) with some modifications. Spray‐infected rosettes of *Arabidopsis* plants (10 days after inoculation with *A. brassicicola*) were ground in liquid nitrogen with a mortar and pestle. To 100 mg frozen plant material, 300 µL lysis buffer [2.5 m LiCl, 50 mm Tris‐HCl, 62.5 mm Na_2_‐ethylenediaminetetraacetic acid (EDTA), 4% Triton X‐100, pH 8.0] and an equal volume of phenol–chloroform–isoamyl alcohol (25 : 24 : 1, v/v) were added, and samples were thoroughly vortexed. After centrifugation (5 min, 16 000 ***g***) at room temperature, the supernatant was recovered and genomic DNA was precipitated by the addition of two volumes of 100% ethanol, incubation for 15 min at −20 °C and another round of centrifugation. The DNA pellet was washed with 70% ethanol, air dried and resuspended in nuclease‐free water. DNA quality and concentration were analysed with a NanoDrop spectrophotometer. Fungal biomass content was determined from total genomic DNA extracts by quantitative real‐time PCR analysis, and the ratio of *A. brassicicola* to *Arabidopsis* genomic DNA was assessed using the same platform and conditions as for the gene expression analyses described above. Primer sequences specific for *A. brassicicola* (Pogány *et al.*, 2009) were derived from the ribosomal *ITS* region of the fungus (forward, 5′‐TCTCCAGTTTGCTGGAGACT‐3′; reverse, 5′‐GGATGCTGACCTTGGCTGGA‐3′), and a primer pair specific for the sequence of *Arabidopsis* *At4g26410* was used to determine plant biomass content in the samples.

### VIGS

TRV, a bipartite virus‐based silencing system, was used for VIGS experiments. TRV1 contains the viral replicase, the RNA‐dependent RNA polymerase and the movement protein, whereas TRV2 contains the coat protein and a multiple cloning site to insert host plant‐derived fragment(s) of target sequences (Hayward *et al*., 2011). pTRV1 (STOCK: CD3‐1039) and pTRV2 in pCAMBIA3301 (STOCK: CD3‐1043) *Agrobacterium* plasmids were obtained from the *Arabidopsis* Biological Resource Center.

First, a marker *GFP* silencing construct (*TRV2‐GFP*) was created to check the efficiency of silencing. For this purpose, a 256‐bp *mGFP* fragment was amplified from pEarlyGate 103 (Earley *et al.*, 2006) with *GFP*‐specific primer pairs (forward, 5′‐CGCTCTAGAATGCCTGAGGGATACGTGCAG‐3′; reverse, 5′‐CGCTCTAGATTCGATGTTGTGGCGGGTCTT‐3′) and inserted into a pGEM^®^‐T Easy vector (Promega, Madison, WI, USA). The *GFP *insert from the purified pGEM^®^‐T Easy vector (Nucleospin Plasmid, Macherey‐Nagel, Düren, NRW, Germany) was digested with *Eco*RI (Thermo Fisher Scientific, Waltham, MA, USA), and the gel‐purified *GFP* fragment (Agarose gel DNA extraction kit, Roche) was ligated into the *Eco*RI‐digested pTRV2 plasmid.

For the *TRV2‐GFP‐PRX* VIGS vector, a 261‐bp cDNA fragment from the *PRX33* gene was amplified (forward, 5′‐CGCGGATCCGCTGATGGCACACAAACATTC‐3′; reverse, 5′‐ GCGGGATCCAATACAATCTGCTCCTGCTCAA‐3′), which shows 31% sequence similarity with *PRX34* cDNA. The target sequence specificity of silencing and avoidance of off‐target gene silencing were controlled and confirmed by the siRNA tool (http://bioinfo2.noble.org/RNAiScan.htm) (Xu *et al.*, 2006) searching its *Arabidopsis thaliana *DCFCI Gene Index V 15 release on 23/04/2010, *Arabidopsis thaliana* TAIR 9 V 9 release on 19/06/2009 and *Arabidopsis thaliana* mRNA (from TIGR Ath1 5). The amplified fragment was cloned into the *Bam*HI restriction site of the TRV2‐GFP vector.

Afterwards, VIGS plasmids were introduced into *Escherichia coli* DH5α by the heat shock method (Tu *et al.*, 2005). The identity of the clones was confirmed by DNA sequence determination. VIGS plasmids were finally purified from *E. coli* (Nucleospin Plasmid, Macherey‐Nagel) and introduced into *Agrobacterium tumefaciens* MOG301 (Hood *et al.*, 1993) by electroporation. For VIGS, *Agrobacterium* strains containing pTRV1 and pTRV2 vectors were grown at 28 °C overnight on LB (Lysogeny Broth) medium supplemented with the appropriate antibiotics (kanamycin sulfate, 30 µg/mL; rifampicin, 50 µg/mL). For inoculum preparation, bacterial cells were suspended in *Agrobacterium* incubation buffer (1.95 g MES (2‐[N‐morpholino]ethanesulfonic acid), 2 g MgCl_2_^.^6H_2_O in 1 L distilled water, pH 5.6) and supplemented with acetosyringone (final concentration of 150 mm). Bacterial cell densities were adjusted with a spectrophotometer to an optical density at 600 nm (OD_600_) = 1.5. After a 3‐h incubation at room temperature, the bacterial suspensions (TRV1 and TRV2), mixed in a ratio of 1 : 1 (v/v), were infiltrated into two lower leaves of 2–3‐week‐old, GFP‐expressing transgenic *Arabidopsis* plants, which were grown at 22 °C in a growth chamber under a 16‐h light/8‐h dark cycle (Burch‐Smith *et al*., 2006).

Three weeks after agroinfiltration, the effect of gene silencing was observed under UV light and gene‐silenced plants were selected and used for mechanical inoculation as viral inoculum on new 2–3‐week‐old, GFP‐expressing transgenic *Arabidopsis* seedlings which were kept under a 14‐h light/10‐h dark cycle. Mechanical inoculation was chosen instead of agroinfiltration because the 16‐h light/8‐h dark photoperiod condition required for agroinoculation did not provide *Arabidopsis* plants appropriate for pathological experiments with *A. brassicicola* (plants grown under these conditions did not develop useful rosettes). Fourteen days after mechanical inoculation, the effect of gene silencing was tested on entire plants under UV light and by real‐time PCR from the UV‐selected plants. The mRNA contents of *PRX33* and *PRX34* were calculated using the same platform and conditions as for the real‐time PCR assays above (*PRX33*: forward, 5′‐AAATTCAGCCCGAGGATTTC‐3′; reverse, 5′‐GAGCAGCAATGGTGAGCATA‐3′; *PRX34*: forward, 5′‐CGAGAAACCATTGTAAATGAGT‐3′; reverse, 5′‐CCGAGCCGAATTTGCG‐3′).

### H_2_O_2_ detection

Mock‐inoculated and *A. brassicicola*‐infected *Arabidopsis* plants (2 days after inoculation) were vacuum infiltrated with DAB (Sigma‐Aldrich, St. Louis, MO, USA) dissolved in distilled water (1 mg/mL), incubated in the light for 2 h, decolorized with plant clearing solution (80% ethanol, 20% chloroform, 0.15% trichloroacetic acid) and mounted in 50% glycerol solution, as described previously (Pogány *et al.*, 2004). Stained plants were examined by light microscopy.

The fluorescent dye DCFH‐DA was also used to detect H_2_O_2_. Detached *Arabidopsis* leaves in the middle position (in positions 5–8) were vacuum infiltrated with 0.4 mm DCFH‐DA in 10 mm sodium phosphate buffer (pH 7.4), as described by Bozsó *et al. *(2005). After a 10‐min incubation period in the dark, leaves were photographed under 302–365‐nm UV light illumination by an AlphaImager Mini gel documentation system (ProteinSimple, Santa Clara, CA, USA). For each genotype or treatment, 30 leaves (in the middle position) were assayed and, for each leaf, an average pixel fluorescence intensity was calculated by AlphaView software of AlphaImager^®^ Systems using the Multiplex Band Analysis function.

### Statistical analysis

Experimental data were analysed using Student’s *t*‐test, the non‐parametric Mann–Whitney *U*‐test or one‐way analysis of variance (ANOVA) and subsequent Tukey’s honestly significant difference test. Statistical analyses were performed with IBM SPSS Statistics 20 Software (Armonk, New York, USA).

## Supporting information


**Fig. S1  **Transcript levels of five polyamine oxidase (*PAO*)‐encoding genes of wild type (Col‐0) *Arabidopsis thaliana*. Five to 6 weeks old *Arabidopsis* plants (whole rosettes) were spray‐inoculated with *A. brassicicola* conidium suspension used in a concentration of 5 x 10^5^ conidia in 1 mL distilled water. Samples of mock‐inoculated and *A. brassicicola*‐infected plants (24 hai) were analyzed. Transcript levels of the five *PAO* isoforms are not increased as a result of the fungal infection. Data represent the mean of three independent biological samples with three technical replicates for each. Statistical analysis was performed using Student's *t*‐test. Asterisks indicate statistically significant difference (**α = 0.01).

**Fig. S2  **Transcript level of apoplastic class III peroxidase gene *PRX33* (*At3g49110*) is reduced in the *Arabidopsis* *prx33* knock‐down T‐DNA insertion line (SALK_062314C). Transcript levels were quantified in untreated *Arabidopsis* plants by real‐time RT‐PCR. The results show the average of two experiments each comprising three biological samples (each sample composed as a pool of 3 *Arabidopsis* rosettes) analyzed in three technical replicates. Statistical analysis was performed using Student's *t*‐test. Asterisks indicate statistically significant difference (***α = 0.001).

**Fig. S3  **Transcript levels of apoplastic class III peroxidase genes *PRX33* (*At3g49110*) and *PRX34 *(*At3g49120*) are reduced in Col‐0 *Arabidopsis* plants that were treated with a VIGS construct (*TRV‐PRX*) that targets *PRX33* and *PRX34* mRNA sequences. Plants labelled as *TRV‐GFP* were treated with the control construct that targets only mRNA sequences of the *GFP* marker gene. Silencing of transgene *GFP* transcript levels and a subsequent abolishment of GFP‐derived green fluorescence in VIGS‐treated plants was used to detect the occurrence of successful gene silencing events. Five to 6 weeks old *Arabidopsis* plants (whole rosettes) were spray‐inoculated with *A. brassicicola* conidium suspension used in a concentration of 5 x 10^5^ conidia in 1 mL distilled water. *PRX33* and *PRX34* mRNA levels were monitored in VIGS‐treated *Arabidopsis* plants before (A) and after (B) inoculation with *A. brassicicola* (24 hai) by real‐time RT‐PCR. The results show the average of two experiments each comprising three biological samples (each sample composed as a pool of 3 *Arabidopsis* rosettes) analyzed in three technical replicates. Statistical analysis was performed using Student’s t‐test. Asterisks indicate statistically significant differences (*α = 0.05, **α = 0.01, ***α = 0.001).

**Fig. S4  **Confirmation of T‐DNA insertion by genotyping *prx33* (SALK_062314C) *Arabidopsis* line. PCR analysis to confirm the presence of T‐DNA was performed using genomic DNA of the *Arabidopsis* insertion line *prx33* (SALK_062314C) and of wild type Col‐0. The first 4 lanes show results of PCR amplifications, where primers specific for *PRX33* sequence (forward 5’ ‐ATTATAGTTGTTGTCAGCATTAGCA‐3’, reverse 5’‐ACCATTTGTTCCTCTGAAGCA‐3’) were used with Col‐0 and *prx33* genomic DNA extracts as templates. The last three lanes exhibit PCR results where a T‐DNA left border primer (*LBa1*:5’‐ TGGTTCACGTAGTGGGCCATCG ‐3’) was combined with *PRX33* sequence specific forward primer using *prx33* insertion line genomic DNA extract confirming the location of T‐DNA insertion. No template controls were included. PCR products were fractionated in 1% agarose gel and DNA was visualized by staining with GelRed (Fremont, California, USA).

**Table S1** *Arabidopsis* T‐DNA insertion lines used in this work.

**Table S2  **Primer sequences used in this work.
