# Supplementary material for: Contribution of cell wall peroxidase‐ and NADPH oxidase‐derived reactive oxygen species to Alternaria brassicicola‐induced oxidative burst in Arabidopsis
Source: Mol Plant Pathol. 2019 Feb 8;20(4):485–99. doi: 10.1111/mpp.12769 (PMC6637864; doi:10.1111/mpp.12769)
Supplement: Supplementary file 1 — Fig. S1 Transcript levels of five polyamine oxidase (PAO)‐encoding genes of wild type (Col‐0) Arabidopsis thaliana. Five to 6 weeks old Arabidopsis plants (whole rosettes) were spray‐inoculated with A. brassicicola conidium suspension used in a concentration of 5 x 10^5^ conidia in 1 mL distilled water. Samples of mock‐inoculated and A. brassicicola‐infected plants (24 hai) were analyzed. Transcript levels of the five PAO isoforms are not increased as a result of the fungal infection. Data represent the mean of three independent biological samples with three technical replicates for each. Statistical analysis was performed using Student's t‐test. Asterisks indicate statistically significant difference (**α = 0.01). [file MPP-20-485-s001.docx]

**Supplemental Fig. S1**. Transcript levels of five polyamine oxidase (*PAO*)-encoding genes of wild type (Col-0) *Arabidopsis thaliana*. Five to 6 weeks old *Arabidopsis* plants (whole rosettes) were spray-inoculated with *A. brassicicola* conidium suspension used in a concentration of 5 x 10^5^ conidia in 1 mL distilled water. Samples of mock-inoculated and *A. brassicicola*-infected plants (24 hai) were analyzed. Transcript levels of the five *PAO* isoforms are not increased as a result of the fungal infection. Data represent the mean of three independent biological samples with three technical replicates for each. Statistical analysis was performed using Student's *t*-test. Asterisks indicate statistically significant difference (**α = 0.01).

**
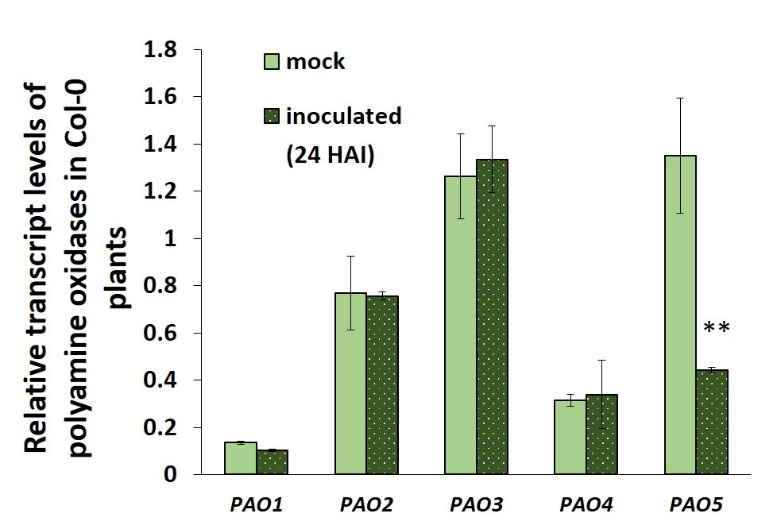
**
