# Supplementary material for: Contribution of cell wall peroxidase‐ and NADPH oxidase‐derived reactive oxygen species to Alternaria brassicicola‐induced oxidative burst in Arabidopsis
Source: Mol Plant Pathol. 2019 Feb 8;20(4):485–99. doi: 10.1111/mpp.12769 (PMC6637864; doi:10.1111/mpp.12769)

**Supplemental Fig. S2.** Transcript level of apoplastic class III peroxidase gene *PRX33* (*At3g49110*) is reduced in the *Arabidopsis* *prx33* knock-down T-DNA insertion line (SALK_062314C). Transcript levels were quantified in untreated *Arabidopsis* plants by real-time RT-PCR. The results show the average of two experiments each comprising three biological samples (each sample composed as a pool of 3 *Arabidopsis* rosettes) analyzed in three technical replicates. Statistical analysis was performed using Student's *t*-test. Asterisks indicate statistically significant difference (***α = 0.001).


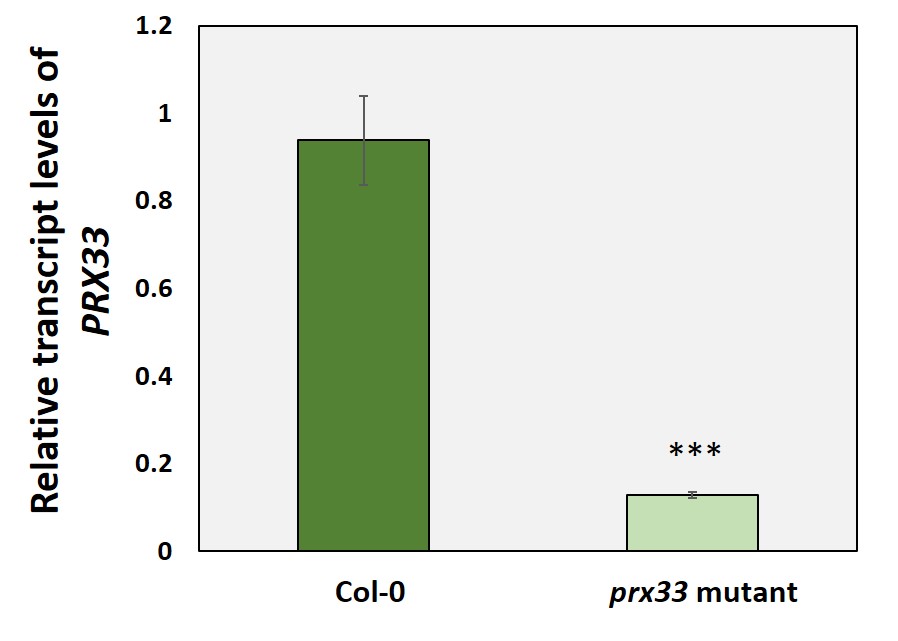

Supplement: Supplementary file 2 — Fig. S2 Transcript level of apoplastic class III peroxidase gene PRX33 (At3g49110) is reduced in the Arabidopsis prx33 knock‐down T‐DNA insertion line (SALK_062314C). Transcript levels were quantified in untreated Arabidopsis plants by real‐time RT‐PCR. The results show the average of two experiments each comprising three biological samples (each sample composed as a pool of 3 Arabidopsis rosettes) analyzed in three technical replicates. Statistical analysis was performed using Student's t‐test. Asterisks indicate statistically significant difference (***α = 0.001). [file MPP-20-485-s002.docx]
