# Supplementary material for: Contribution of cell wall peroxidase‐ and NADPH oxidase‐derived reactive oxygen species to Alternaria brassicicola‐induced oxidative burst in Arabidopsis
Source: Mol Plant Pathol. 2019 Feb 8;20(4):485–99. doi: 10.1111/mpp.12769 (PMC6637864; doi:10.1111/mpp.12769)
Supplement: Supplementary file 3 — Fig. S3 Transcript levels of apoplastic class III peroxidase genes PRX33 (At3g49110) and PRX34 (At3g49120) are reduced in Col‐0 Arabidopsis plants that were treated with a VIGS construct (TRV‐PRX) that targets PRX33 and PRX34 mRNA sequences. Plants labelled as TRV‐GFP were treated with the control construct that targets only mRNA sequences of the GFP marker gene. Silencing of transgene GFP transcript levels and a subsequent abolishment of GFP‐derived green fluorescence in VIGS‐treated plants was used to detect the occurrence of successful gene silencing events. Five to 6 weeks old Arabidopsis plants (whole rosettes) were spray‐inoculated with A. brassicicola conidium suspension used in a concentration of 5 x 10^5^ conidia in 1 mL distilled water. PRX33 and PRX34 mRNA levels were monitored in VIGS‐treated Arabidopsis plants before (A) and after (B) inoculation with A. brassicicola (24 hai) by real‐time RT‐PCR. The results show the average of two experiments each comprising three biological samples (each sample composed as a pool of 3 Arabidopsis rosettes) analyzed in three technical replicates. Statistical analysis was performed using Student’s t‐test. Asterisks indicate statistically significant differences (*α = 0.05, **α = 0.01, ***α = 0.001). [file MPP-20-485-s003.docx]

**Supplemental Fig. S3**. Transcript levels of apoplastic class III peroxidase genes *PRX33* (*At3g49110*) and *PRX34* (*At3g49120*) are reduced in Col-0 *Arabidopsis* plants that were treated with a VIGS construct (*TRV-PRX*) that targets *PRX33* and *PRX34* mRNA sequences. Plants labelled as *TRV-GFP* were treated with the control construct that targets only mRNA sequences of the *GFP* marker gene. Silencing of transgene *GFP* transcript levels and a subsequent abolishment of GFP-derived green fluorescence in VIGS-treated plants was used to detect the occurrence of successful gene silencing events. Five to 6 weeks old *Arabidopsis* plants (whole rosettes) were spray-inoculated with *A. brassicicola* conidium suspension used in a concentration of 5 x 10^5^ conidia in 1 mL distilled water. *PRX33* and *PRX34* mRNA levels were monitored in VIGS-treated *Arabidopsis* plants before (A) and after (B) inoculation with *A. brassicicola* (24 hai) by real-time RT-PCR. The results show the average of two experiments each comprising three biological samples (each sample composed as a pool of 3 *Arabidopsis* rosettes) analyzed in three technical replicates. Statistical analysis was performed using Student’s t-test. Asterisks indicate statistically significant differences (*α = 0.05, **α = 0.01, ***α=0.001).

**
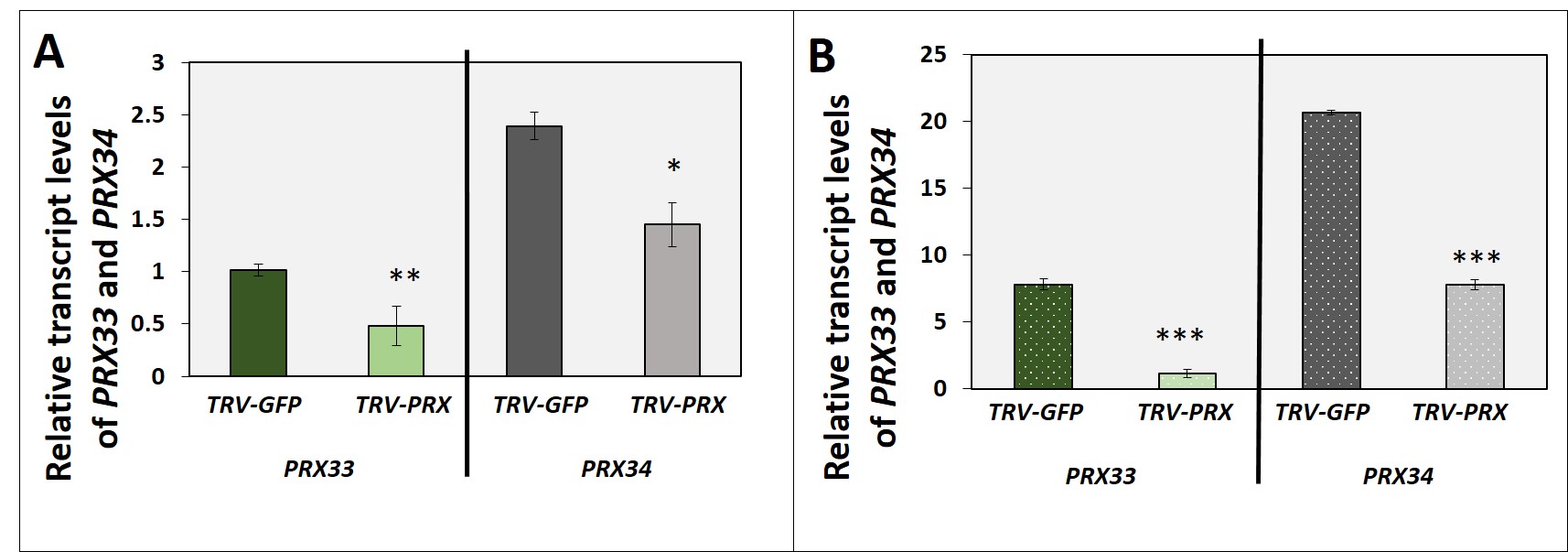
**
