# Supplementary material for: Contribution of cell wall peroxidase‐ and NADPH oxidase‐derived reactive oxygen species to Alternaria brassicicola‐induced oxidative burst in Arabidopsis
Source: Mol Plant Pathol. 2019 Feb 8;20(4):485–99. doi: 10.1111/mpp.12769 (PMC6637864; doi:10.1111/mpp.12769)

**Supplemental Fig. S4.** Confirmation of T-DNA insertion by genotyping *prx33* (SALK_062314C) *Arabidopsis* line. PCR analysis to confirm the presence of T-DNA was performed using genomic DNA of the *Arabidopsis* insertion line *prx33* (SALK_062314C) and of wild type Col-0. The first 4 lanes show results of PCR amplifications, where primers specific for *PRX33* sequence (forward 5’ -ATTATAGTTGTTGTCAGCATTAGCA-3’, reverse 5’-ACCATTTGTTCCTCTGAAGCA-3’) were used with Col-0 and *prx33* genomic DNA extracts as templates. The last three lanes exhibit PCR results where a T-DNA left border primer (*LBa1*:5’- TGGTTCACGTAGTGGGCCATCG -3’) was combined with *PRX33* sequence specific forward primer using *prx33* insertion line genomic DNA extract confirming the location of T-DNA insertion. No template controls were included. PCR products were fractionated in 1% agarose gel and DNA was visualized by staining with GelRed. (Fremont, California, USA).


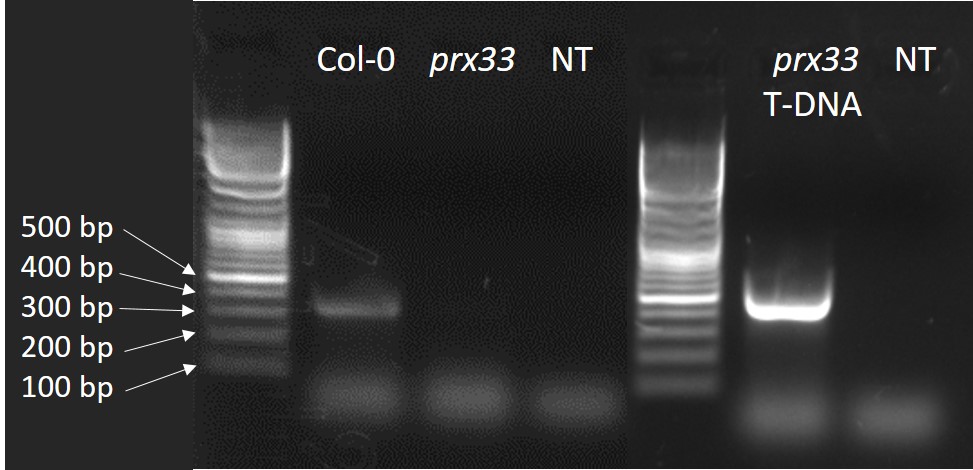

Supplement: Supplementary file 4 — Fig. S4 Confirmation of T‐DNA insertion by genotyping prx33 (SALK_062314C) Arabidopsis line. PCR analysis to confirm the presence of T‐DNA was performed using genomic DNA of the Arabidopsis insertion line prx33 (SALK_062314C) and of wild type Col‐0. The first 4 lanes show results of PCR amplifications, where primers specific for PRX33 sequence (forward 5’ ‐ATTATAGTTGTTGTCAGCATTAGCA‐3’, reverse 5’‐ACCATTTGTTCCTCTGAAGCA‐3’) were used with Col‐0 and prx33 genomic DNA extracts as templates. The last three lanes exhibit PCR results where a T‐DNA left border primer (LBa1:5’‐ TGGTTCACGTAGTGGGCCATCG ‐3’) was combined with PRX33 sequence specific forward primer using prx33 insertion line genomic DNA extract confirming the location of T‐DNA insertion. No template controls were included. PCR products were fractionated in 1% agarose gel and DNA was visualized by staining with GelRed (Fremont, California, USA). [file MPP-20-485-s004.docx]
