# Supplementary material for: Contribution of cell wall peroxidase‐ and NADPH oxidase‐derived reactive oxygen species to Alternaria brassicicola‐induced oxidative burst in Arabidopsis
Source: Mol Plant Pathol. 2019 Feb 8;20(4):485–99. doi: 10.1111/mpp.12769 (PMC6637864; doi:10.1111/mpp.12769)
Supplement: Supplementary file 5 — Table S1 Arabidopsis T‐DNA insertion lines used in this work. [file MPP-20-485-s005.docx]

**Supplemental Table S1.** *Arabidopsis* T-DNA insertion lines used in this work.

| **T-DNA insertion line** | **Gene name** | **AGI code** | **Stock name** |
| --- | --- | --- | --- |
| *prx33* | *Peroxidase 33* | *At3g49110* | SALK_062314C |
| *prx34* | *Peroxidase 34* | *At3g49120* | SALK 051769C |
| *rbohd* | *Respiratory Burst Oxidase Homologue D* | *At5g47910* | SALK_070610C |
|  |  |  |  |
| *jar1* | *Jasmonate Resistant 1* | *At2g46370* | SALK_030821C |
| *nho1* | *Nonhost Resistant to P. S. Phaseolicola 1* | *At1g80460* | SALK_067205C |
| *npr1-1* | *Nonexpressor of PR Genes 1* | *At1g64280* | CS3726 |
| *mpk6* | *MAP Kinase 6* | *At2g43790* | SALK_004221C |
| *nia2* | *Nitrate Reductase 2* | *At1g37130* | SALK_088070C |
| *ein2* | *Ethylene Insensitive 2* | *At5g03280* | SALK_086500C |
| *bik1* | *Botrytis-Induced Kinase 1* | *At2g39660* | SALK_005291C |
|  |  |  |  |
| *erecta* | Quantitative Resistance to Plectosphaerella 1 | *At2g26330* | SALK_04410  SALK_066455C |
| *utr3* | *UDP-Galactose Transporter 3* | *At1g14360* | SALK_064051C  SALK_136447C |
| *ann1* | *Annexin 1* | *At1g35720* | SALK_132169C  SALK_149772C |
| *th7* | *Thioredoxin H-type 7* | *At1g59730* | SALK_018227 |
| *cam4* | *Calmodulin 4* | *At1g66410* | SALK_021224 |
| *gstu19* | *Glutathione S-Transferase TAU 19* | *At1g78380* | SALK_041942  SALK_077958C |
| *dwarf1* | *Cell Elongation Protein* | *At3g19820* | SALK_006932  SALK_030692 |
| *arfc1* | *ADP-Ribosylation Factor C1* | *At3g22950* | SALK_027967  SALK_015199C |
| *mapr3* | *Membrane-Associated Progesterone Binding Protein 3* | *At3g48890* | SALK_056475C  SALK_042103C |
| *n/a* | *N/A* | *At4g37445* | SALK_022186C  SALK_135593C |
| *nhl3* | *NDR1/HIN1-like 3* | *At5g06320* | SALK_150318C  SALK_035427C |
