# Supplementary material for: Contribution of cell wall peroxidase‐ and NADPH oxidase‐derived reactive oxygen species to Alternaria brassicicola‐induced oxidative burst in Arabidopsis
Source: Mol Plant Pathol. 2019 Feb 8;20(4):485–99. doi: 10.1111/mpp.12769 (PMC6637864; doi:10.1111/mpp.12769)
Supplement: Supplementary file 6 — Table S2 Primer sequences used in this work. [file MPP-20-485-s006.docx]

**Supplemental Table S2.** Primer sequences used in this work.

| **Name** | **Amplified locus** | **Forward primer (5'-3')** | **Reverse primer (5'-3')** |
| --- | --- | --- | --- |
| ***At4g26410*** | *At4g26410* | GAGCTGAAGTGGCTTCCATGAC | GGTCCGACATACCCATGATCC |
| ***RbohD*** | *At5g47910* | CTGGACACGTAAGCTCAGGA | GCCGAGACCTACGAGGAGTA |
| ***PRX33*** | *At3g49110* | AAATTCAGCCCGAGGATTTC | GAGCAGCAATGGTGAGCATA |
| ***PRX34*** | At3g49120 | CGAGAAACCATTGTAAATGAGT | CCGAGCCGAATTTGCG |
| ***PAO1*** | At5g13700 | CCAGGAGACGATGAAAGAGG | GTAGCTACCGCGTTGAAACC |
| ***PAO2*** | At2g43020 | AGTCATTGCTGTCCCTCTTG | CCTAGGTCGTTGATTGCTTCT |
| ***PAO3*** | At3g59050 | AGTCAGGGATGAGCAGGA | GAGAGAGTGTTGATTACAGGGCGATA |
| ***PAO4*** | At1g65840 | TGTTATTGTGATTGGTAGTGGTA | TAAGGGATTCTCATCAGAGA |
| ***PAO5*** | At4g29720 | TCAGGCAAAGGAGGACTAAAC | CGAGTGGCAGAGTAATGAAGTGA |
| ***A. brassicicola* *ITS*** |  | TCTCCAGTTTGCTGGAGACT | GGATGCTGACCTTGGCTGGA |
| ***GFP*** |  | CGCTCTAGAATGCCTGAGGGATACGTGCAG | CGCTCTAGATTCGATGTTGTGGCGGGTCTT |
| ***PRX33 VIGS*** |  | CGCGGATCCGCTGATGGCACACAAACATTC | GCGGGATCCAATACAATCTGCTCCTGCTCAA |
| ***LBa1* reverse** |  |  | TGGTTCACGTAGTGGGCCATCG |
| ***PRX33* for genotyping SALK_062314C** |  | ATTATAGTTGTTGTCAGCATTAGCA | ACCATTTGTTCCTCTGAAGCA |
